# Biological Characterization, Properties, and Clinical Use of a Novel Homeopathic Antiseptic Oral Recovery Kit: A Narrative Review

**DOI:** 10.3290/j.ohpd.b3630405

**Published:** 2022-11-30

**Authors:** Nathan E. Estrin, Georgios E. Romanos, Walter Tatch, Michael Pikos, Richard J. Miron

**Affiliations:** a Practicing Periodontist, Adjunct Professor, Lake Erie College of Osteopathic Medicine, School of Dental Medicine, Bradenton, Florida, USA. Conceptualization, formal analysis, investigation, resources, prepared original draft, read and agreed to the published version of the manuscript.; b Professor, Department of Periodontology, Laboratory for Periodontal-, Implant-, Phototherapy (La-PIP), School of Dental Medicine, Stony Brook University, Stony Brook, NY, USA. Validation, reviewed and edited the manuscript, read and agreed to the published version of the manuscript.; c Practicing Oral Surgeon, Northshore Center for Oral and Facial Surgery and Implantology, Gurnee, IL, USA. Validation, reviewed and edited the manuscript, read and agreed to the published version of the manuscript.; d Practicing Oral Surgeon and Lecturer, Pikos Institute, Tampa Bay, FL, USA. Validation, reviewed and edited the manuscript, supervision, read and agreed to the published version of the manuscript.; e Research Associate, Department of Periodontology, University of Bern, Bern, Switzerland. Conceptualization, formal analysis, investigation, resources, prepared original draft, supervision, project administration, read and agreed to the published version of the manuscript.

**Keywords:** antiseptic solution, cell viability, chlorhexidine, oral rinses, periodontal disease, StellaLife, wound healing

## Abstract

**Summary:** Most available antiseptic solutions have strong antibacterial effects, but many also possess major cytotoxic effects on gingival fibroblasts, osteoblasts, osteoprogenitor cells, and/or epithelial cells. A novel VEGA Oral Care Recovery Kit (StellaLife) consisting of 16 active ingredients that are monographed in the Homeopathic Pharmacopeia of the United States (HPUS) has gained tremendous momentum as a replacement for more cytotoxic oral rinses such as chlorhexidine. While accumulating evidence has thus far supported its use, little of the gathered data have fully described the properties of the oral formulation. Therefore, the aim of the present review article was 3-fold. First, a biological characterization regarding the active ingredients found in StellaLife Recovery Kit including their biological properties was assessed in 4 predominant categories; 1) antimicrobial resistance, 2) accelerated wound healing, 3) pain management control, and 4) anti-cancer properties. The second aim of this review article was to assess both fundamental and clinical research to date comparing VEGA oral rinse (StellaLife) to the more commonly utilized CHX for differences regarding their effect on decreasing bacterial loads as well as cell viability, survival, proliferation, and expression of both regenerative cytokines and inflammatory markers. Lastly, clinical case examples are presented describing the use of StellaLife remedies in a variety of clinical situations. These include but are not limited to wisdom-tooth extraction, extraction site management, dental implants and ridge augmentation, soft-tissue grafting procedures, frenectomies, and also temporary relief of dry sockets, dry mouth, aphthous ulcers, mucositis, lichen planus, among others. In summary, findings from the present review article provide evidence from basic laboratory experiments that validate clinical studies supporting the use of the StellaLife oral rinse regarding its superior biocompatibility and wound healing properties when compared to common antiseptic solutions such as CHX.

Dental plaque consists of a biofilm with a glycocalyx extracellular matrix^21,33^ that has been shown to mimic human tissue, as the aggregate of microbial cells forms a collectively functioning structure.^ 19^ It can be altered by a variety of factors, including dietary habits, stress, smoking, medications, and oral home care.^61,66,69^ Given that the oral cavity is the gateway to the rest of the body, plaque from the oral cavity has been linked to various systemic conditions.^61,69^ Thus, limiting the amount and type dental plaque is important in all fields of dentistry to aid in the prevention of post-surgical infection, caries, and periodontal disease, as well as to promote the overall health of the patient.^19,33,34^

Antimicrobial rinses are commonly employed in oral surgery to decrease bacterial loads and mitigate the risk of contamination after surgery.^13,47^ Nevertheless, their use, despite being highly effective against oral bacteria, has also proven toxic to human cells found in the oral cavity.^59,60^ Previously, our group found that commonly utilized antiseptic solutions, including 1) 0.5% povidone iodine (PI); 2) 0.2% chlorhexidine digluconate (CHX); 3) 1% hydrogen peroxide (H_2_O_2_); and 4) 0.25% sodium hypochlorite (HYP). Each led to a minimum of 50% cell destruction within 10 minutes of rinsing autogenous bone.^59,60^ Thus, many countries, such as Japan, have entirely banned certain products such as CHX as oral rinses due to not only their increases in oral cell death, but also potential increases in additional post-operative inflammation and pain.^23^

Owing to the above-mentioned challenges, more natural herbal compounds have recently been investigated as potential therapeutic options with both the ability to decrease bacterial loads and also promote instead of impair wound healing.^36,46,65^ In contrast to commonly used antimicrobial oral rinses, the efficacy against oral bacteria from herbal extract-based alternatives primarily lies in enhancing wound healing, particularly in conjunction with oral surgical treatment.^36^ This alternative and more homeopathic approach to pharmacological oral care has been grounded upon a well-established body of literature presented in this article, suggesting that currently used antimicrobial chemotherapeutics, such as chlorhexidine, can have quite negative effects on cellular components of wound healing,^23,46^ and may potentially be the cause of significant post-operative pain.

In the United States, opioids are commonly prescribed by dentists for the management of acute pain after oral surgery, but their use has been questioned in recent years based on the number of teenagers and young adults who have reported that their drug abuse/addictions to other narcotics often began with opioids prescribed by dentists. Since the mid 1990s, deaths caused specifically from opioid overdose has more than quadrupled in the US, which parallels precisely the increase in opioid prescriptions issued in dental and medical practices.^48,53^ Tatch^68^ has clearly shown that opioid prescription can be reduced in oral surgery without compromising pain management. In a 3-year retrospective study, a 3-fold reduction in heavy opioid prescription was found in an oral surgery practice when the StellaLife Recovery kit was utilized.^68^ While pain management is needed post-surgery, questions have arisen regarding the use of common antiseptic solutions that are known to cause additional inflammation (such as CHX).

One recent, novel, opioid-free homeopathic option that has gained tremendous momentum as an oral rinse is VEGA Oral Care Recovery Kit (Fig 1), which has been recognized as an effective treatment to reduce pain post-procedure in several clinical studies. It consists of 16 active ingredients that are monographed in the Homeopathic Pharmacopeia of the United States (HPUS) and recognized for their accelerated healing properties with antimicrobial properties.^38^ Briefly, Lee and Suzuki^38^ utilized the StellaLife recovery kit to evaluate pain, wound healing, edema and ecchymosis in two groups of patients, one with opioid use and the other without, who underwent harvesting of monocortical blocks of bone from the posterior mandible and ramus under local anesthesia. The authors reported that this novel, opioid-free alternative form of therapy administered pre-emptively prior to block bone-graft surgery resulted in decreased postoperative pain, and less reliance on opioid analgesics.^38^ Furthermore, Tatch^68^ also found a 3-fold reduction in prescribed opioids when the StellaLife recovery kit was added to routine private practice procedures over a 3-year period (>1000 patients evaluated, with reduction in opioids prescribed from 59% down to 19% of patients).^68^ A single application of StellaLife oral rinse was reported to significantly reduce commonly found anaerobic bacteria, including *Streptococcus mutans, Actinomyces viscosus, Streptococcus pyogenes, Porphyromonas gingivalis,* and* Bacteroides fragilis*. Therefore, with accumulating evidence supporting its use, the aim of this review article is 3-fold.

**Fig 1:**
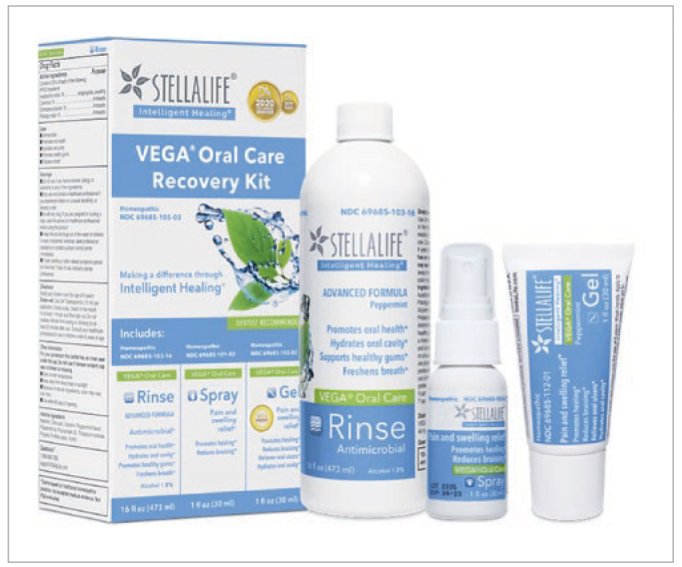
StellaLife VEGA Oral Care Recovery kit; available as a rinse, gel, or spray.

First, a biological characterization regarding the active ingredients found in StellaLife Recovery Kit including its biological properties was assessed in 4 predominant categories; 1) antimicrobial resistance, 2) accelerated wound healing, 3) pain management control and 4) anti-cancer properties. Second, fundamental and clinical research to date comparing StellaLife Oral Rinse to the more commonly utilized CHX was assessed for differences regarding its effectiveness in decreasing bacterial loads as well as its effect on gingival fibroblast and epithelial cell viability, survival, proliferation and expression of both regenerative cytokines as well as inflammatory markers. Lastly, clinical case examples are presented describing the use of StellaLife in a variety of clinical situations. These include but are not limited to wisdom-tooth extraction, extraction site management, dental implants and ridge augmentation, soft-tissue grafting procedures, frenectomies, and also temporary relief of dry sockets, dry mouth, aphthous ulcers, mucositis, lichen planus, i.a. (Fig 1).

## Biological Characterization and Properties of Stellalife

### Antimicrobial Resistance

*Azadirachta indica* (neem), while a major ingredient in StellaLife, was first utilized in Siddha medicine, India’s ancient medical system.^29^ Its nicknames include “Nature’s Drug Store”, “Village Pharmacy”, and “Heal All”.^29,31,50^ In addition, in 2012 it was named “Tree of the 21st century” by the United Nations based on its wound healing characteristics and antimicrobial properties.^29^ Its various applications in medicine are based on the fact that neem contains over 300 different phytochemicals.^29^ Neem has been shown to possess anti-fungal, antipyretic, antihistamine, antiseptic, and anti-inflammatory properties.^10,29^ While the leaves, flowers, oil, and bark have all been utilized in medicine, the twigs have previously been successfully utilized for cleaning teeth in millions of patients.^29^ Neem has therefore been widely utilized in the oral cavity much longer than modern toothpaste and oral home-care products.

Pre-clinical research has also supported its use. An in-vitro study by Sarkar et al^57^ compared the effects of nimbolide, a phytochemical isolate from neem, against methicillin-resistant *S. aureus* (MDR MRSA). Nimbolide exhibited high inhibitory activity against MDR MRSA with a minimum inhibitory concentration (MIC) and minimal bactericidal concentration (MBC) lower than that of common antibiotics, including ciprofloxacin, tetracycline, and streptomycin.^57^ Additionally, no cytotoxicity to human erythrocytes was shown.^57^ It was found that nimbolide altered the bacterial cell-membrane structure and permeability, even to the extent of complete disintegration of the bacterial envelopes, causing cell lysis.^57^

Elavarasu et al^21^ also investigated the antibacterial effects of neem. In that study, plaque samples were collected and cultured from five periodontally affected patients.^21^ Those authors observed a reduction in microorganisms by the formation of inhibition zones on agar plates. In a randomized controlled clinical trial,^11^ the effects of an *Azadirachta indica*-based mouthrinse were compared with standard 0.12% chlorhexidine rinse. In that study, 54 patients were instructed to rinse for 7 days; plaque index, gingival index, gingival bleeding and counts of *S. mutans* in salivary samples were taken.^11^ Both groups revealed a significant reduction in clinical and microbiological parameters with no significant differences reported between groups in any of the measured clinical parameters.^11^

Another active ingredient in StellaLife, *Calendula*, has also been shown to have antimicrobial properties.^15,35^ *Calendula officinalis*, also known as “Pot Marigold”, is a plant native to the Mediterranean area, and has yellow and orange flowers.^15,35^ The plant is rich in flavonoids, glycosides, sterols, tannins, and essential oils.^15,35^ *Calendula* extracts have been shown to possess antibacterial activity against both gram-negative and gram-positive bacteria^15^ with anti-fungal properties towards at least 23 fungi.^24^ In a randomized controlled trial by Khairnar et al,^35^ 240 gingivitis patients rinsed twice a day for 6 months with either diluted *Calendula* or a placebo. Clinical parameters were recorded at baseline, 3 and 6 months with professional scaling conducted at 3 months.^35^ The *Calendula* group showed a significant reduction in plaque index (PI), gingival index (GI), and sulcus bleeding index (SBI) at both the 3- and 6-month visits, whereas the control group only showed a significant reduction at 6 months following the professional cleaning.^35^ In a similar study,^2^ which included 30 patients, *Calendula* was compared with *Plantago* (also an active ingredient in StellaLife). All patients included had gingivitis and were randomly assigned to rinse twice a day with either *Calendula*- or *Plantago*-based rinses.^1^ GI, BI, and SBI were recorded at baseline, 3 and 6 months, but unlike the previous study by Khairnar et al,^35^ professional scaling was only done at the initial visit.^1^ While both groups showed a significant reduction in all clinical parameters at the 3 and 6 months, *Calendula* showed a statistically significantly greater reduction in plaque and gingivitis scores.^1^

Other active ingredients in StellaLife have shown antimicrobial and antivital properties. *Plantago*, known for its antimicrobial properties, has also been shown to possess anti-viral and immunomodulary activity.^16^ Propolis, which is a composite of beeswax and pollen produced by honey bees, has also been shown to possess antifungal and antimicrobial properties against common periodontopathic bacteria, among others.^25^ While the active ingredients mentioned, as well as others found in StellaLife, have been shown to exhibit antibacterial, antifungal, and antiviral properties, additional studies are necessary in order to evaluate their synergy in StellaLife, as well as various combination approaches when compared to monotherapies.

### Accelerated Healing

While most rinses such as CHX are very strong antimicrobials, several studies have also demonstrated their harmful and cytotoxic effects on human cells.^98^ As discussed in the previous section, several ingredients in StellaLife such as *Calendula* and neem are selectively cytotoxic to bacteria but have been shown to cause no harm to human cells.

*Plantago major,* for instance, an active ingredient in StellaLife, has been used for medicinal purposes for centuries to treat abscesses, acne, burns, and many other injuries.^56^ In studies by Zubair et al,^72,73^ the wound healing properties of *Plantago* were evaluated in a scratch-wound assay and a wound-healing model. It was concluded that *Plantago major* exhibited wound healing potential in a dose-dependent manner in a porcine skin wound-healing model.^72^ Additionally, cell migration and proliferation was improved in the scratch-wound model and several cell culture assays.^71^ The wound healing properties of StellaLife also exhibited similar properties demonstrated in a study by Zhou et al,^71^ discussed later in this article.

*Arnica montana*, also a StellaLife ingredient, is native to Siberia and Central Europe and has been utilized in StellaLife owing to its anti-inflammatory and wound healing properties.^3,42^ In an in-vitro study, arnica was shown to help stimulate gene expression in macrophages towards their wound-healing M2 phenotype.^42^ While many of these active ingredients in StellaLife have been shown to possess accelerated wound healing individually, studies specifically investigating StellaLife have also shown excellent wound healing properties, especially when compared to CHX.

### Pain Management

Pain management is a critical property of antiseptic solutions, especially in terms of trying to limit opioid use in the US. Perception of pain is initiated in the peripheral nervous system (PNS) before the central nervous systems (CNS) is involved, when nociceptors transmit signals along small myelinated A and unmyelinated C fibers before synapsing in the dorsal horn of the spinal cord.^27,53^ Signals are then relayed to the thalamus and cortex via the spinothalamic tract of the spinal cord.^28^ Psychological variables are also known to modulate activity in the dorsal horn.^27^ Active ingredients in StellaLife such as *Aconitum, Gelsemium,* and *Ignatia* have shown anti-anxiolytic properties.^9,40,43^ This is critically important in dentistry, since patients exhibiting anxiety or depression experience more pain from surgery.^67^ Thus, pain management is a critical factor in North American society, where the goal is to manage patient expectations and deliver fast recovery periods with little to no postoperative pain.

Pain from surgical insult occurs in two phases: an initial phase exhibiting acute pain at the point of noxious stimuli (or incision), and a second phase of prolonged, dull pain around the stimulated area.^27^ It has long been proposed that the pain stimulus is initiated by inflammatory mediators released at the site of surgery.^18,27^ The goal of both pre-emptive and post-operative analgesia is to prevent the increase in inflammatory mediators post-surgery.^27,49^ Unlike most pain management regimes, minimizing post-operative pain with StellaLife begins 3 days pre-surgically.

VEGA Oral Care Recovery Kit by StellaLife has 16 active homeopathic ingredients including *Arnica*, chamomile, and *Aconitum*. In a study evaluating the mechanisms of *Arnica montana* flower methanol extract (AMME) in an arthritic rat model,^64^ the authors showed that AMME significantly reduced the amount of oxygen free-radicals and pro-inflammatory cytokines such as TNF-α, IL-1, and IL-6, without any toxic response in the host. Interestingly, when compared to the commonly utilized corticosteroid dexamethasone, AMME showed greater therapeutic efficacy.^63^

Another ingredient in StellaLife is chamomile, which is commonly utilized alone for pain management due to its anti-inflammatory and anti-nociceptive properties.^44,50^ The mechanism of action is believed to be associated with its ability to inhibit pro-inflammatory cytokines such as TNF-α, IL-1, IL-6 and IL-8,^44^ and by its inhibition of COX, a main participant in nociception and inflammation.^ 50^ Chamomile (*Matricaria recutita*), which has a history use as a topical anesthetic, may also function as a selective cycloxegenase (COX)-2 inhibitor.^ 50^ It has also been suggested to have a synergistic effect when employed in combination with other non-steroidal anti-inflammatory drugs (NSAIDs), such as diclofenac.^ 50^ Chamomile has also been shown to reduce sodium channels in a dose-dependent fashion, thus decreasing peripheral nerve excitability.^6^ In addition to having anti-anxiolytic properties, *Aconitum* has been shown to possess anti-nociceptive properties, also via blocking voltage-dependent sodium channels.^70^ Thus, it was used in ancient Chinese and Japanese medicine as an analgesic.^5^

In summary, many of the active ingredients in StellaLife exhibit anti-inflammatory and anti-nociceptive properties that aid in pain management. Pathways involved include the reduction of pro-inflammatory cytokines, COX inhibition, anti-anxiolytics, and the blockade of neuronal sodium currents. While many of our patients report a soothing effect when using the StellaLife rinse and gel, more research is necessary to evaluate how these ingredients work synergistically to manage pain control from a pre-emptive and post-operative point of view.

### Anti-Cancer Properties

**Fig 2:**
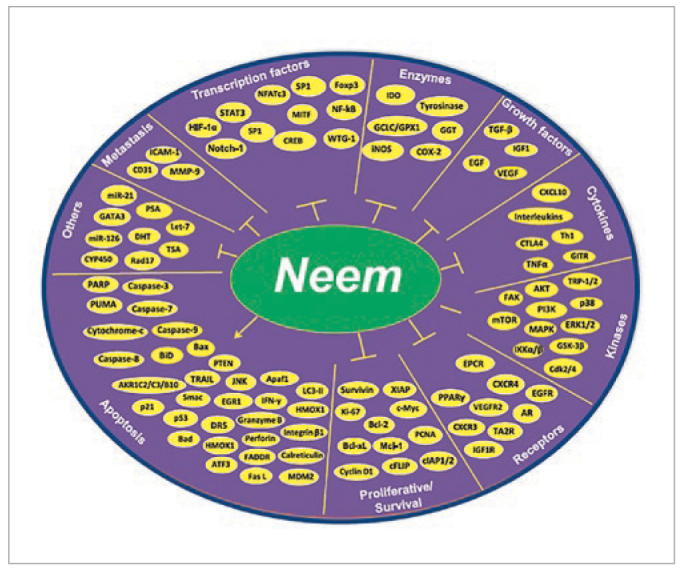
Demonstration of the common molecular targets of neem and its constituents. Figure reprinted with permission from Gupta et al, 2017.

Cancer cells are characterized by their uncontrolled growth, proliferation, induction of angiogenesis, and resistance to normal immunological responses.^31^ Given that cancer is a complex disease involving multiple cell-signaling pathways, mono-targeted therapies are limited in their effectiveness in oncology patients.^28,58^ Given the numerous phytochemicals derived from neem (over 300 identified) and the large number of molecular targets (Fig 2), it represents a multi-factorial strategy for inhibiting and preventing cancer cells.^28,41^

Nimbolide, one of the triterpenoids isolated from the flowers and leaves of neem, has been shown to inhibit proliferation of numerous types of tumor cells.^28^ One action of nimbolide is its role inhibiting NF-kB activation, which plays a critical role in tumor cell proliferation, invasion, and angiogenesis.^2^^,29^ NF-kB is a transcription factor that is activated in response to both carcinogens, such as DMBA, and pro-inflammatory cytokines, such as TNF-a, IL-1 and IL-6.^28^ These cytokines also play a critical role in the development of periodontitis.^30^

Furthermore, a prominent feature of cancer cells is their resistance to apoptosis, a fundamental part of cell regulation and tissue homeostasis.^39^ In cancer cells, reduced apoptosis has been shown to be due to an imbalance in the Bcl-2:Bax ratio, a family of proteins responsible for the intrinsic apoptotic pathway.^39^ In a study by Manikandan et al,^41^ anti-cancerous effects of neem leaf extracts were evaluated in DMBA-induced hamster buccal pouch (HBP) carcinogenesis. They found that higher concentrations of neem leaf enhanced the chemo-preventative efficacy against cancer cells.^41^

Several other studies have also demonstrated anti-cancerous properties of neem-tree derivatives.^31,51^ Neem-seed oil demonstrated apoptosis in human breast cancer cells (HBCCs) by inducing apoptosis.^61^ While research is ongoing for neem extracts and their anti-cancer properties, more clinical research is necessary to further evaluate how StellaLife may improve care, regarding the growing number of oral cancer cases found worldwide.

### Biological Properties of StellaLife Oral Rinse vs Chlorhexidine

Chlorhexidine (CHX) has historically been the gold standard in terms of oral rinses used post-operatively in dentistry. Nevertheless, previous studies have highlighted the potential harmful effects of CHX on wound healing in the oral cavity (especially towards gingival fibroblasts).^47^

**Fig 3:**
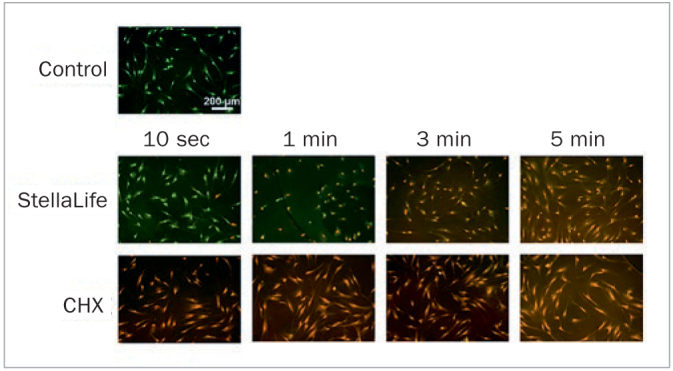
Cell viability of human gingival fibroblasts (HGF-1 cells) exposed to 100% of antiseptic oral rinses (either StellaLife or CHX) for 10 s, 1 min, 3 min, or 5 min respectively. Live-dead staining was done with viable cell appearing in green and dead cells in red. StellaLife showed viable cells at 10 s and 1 min treatments, whereas cells cultured with CHX demonstrated apoptotic cells in as little as 10 s. Reprinted with permission from Fujioka-Kobayashi, 2020.

**Fig 4:**
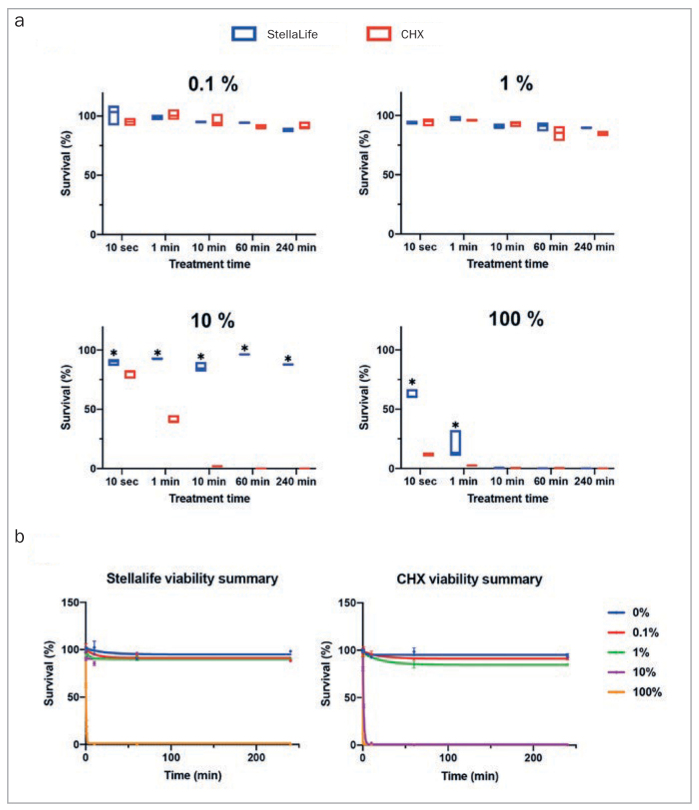
Cell viability of HGF-1 cells exposed to 0.1%, 1%, 10% or 100% of antiseptic oral rinses (either StellaLife or CHX) for 10 s, 1 min, 10 min, 60 min, and 240 min. (a) Two antiseptic oral rinses at each dilution were compared for cell survival properties. *Denotes statistically significantly higher than the other treatment modality, p < 0.05. (b) The effect of dilutions of antiseptic oral rinses on cell survival were summarized. In general, 0.1% and 1% antiseptic oral rinses did not affect cell viability. Reprinted with permission from Fujioka-Kobayashi, 2020.

As such, in a study by Fujioka-Kobayashi et al,^23^ StellaLife was compared to CHX in-vitro on gingival fibroblast viability, survival at various concentrations, migration assay, proliferation activity, as well expression of both regenerative growth factors and inflammatory markers. In a first experiment, the effects of both antiseptic oral rinses were investigated on the viability of human gingival fibroblasts (HGF-1 cells). It was observed that a greater number of viable cells were reported in the StellaLife group (green cells) when compared to CHX (even at 10 s) (Fig 3). Upon further dilutions, StellaLife also demonstrated statistically significantly higher cell survival when compared to CHX, especially at dilutions of 10% and 1% initial concentrations (i.e. 0.02% CHX and 0.002% CHX, respectively) (Fig 4a). Most notably, a 1-min rinse with a 10% CHX concentration resulted in >50% cell death, and by 10 min, 100% of cells were no longer viable. In contrast, cells exposed to 10% StellaLife demonstrated 100% cell survival up to 240 min (Fig 4a). Since cell survival remained high for both antiseptic oral rinses at a 1% concentration, the 1% dilution was therefore chosen for further cell assays.

**Fig 5:**
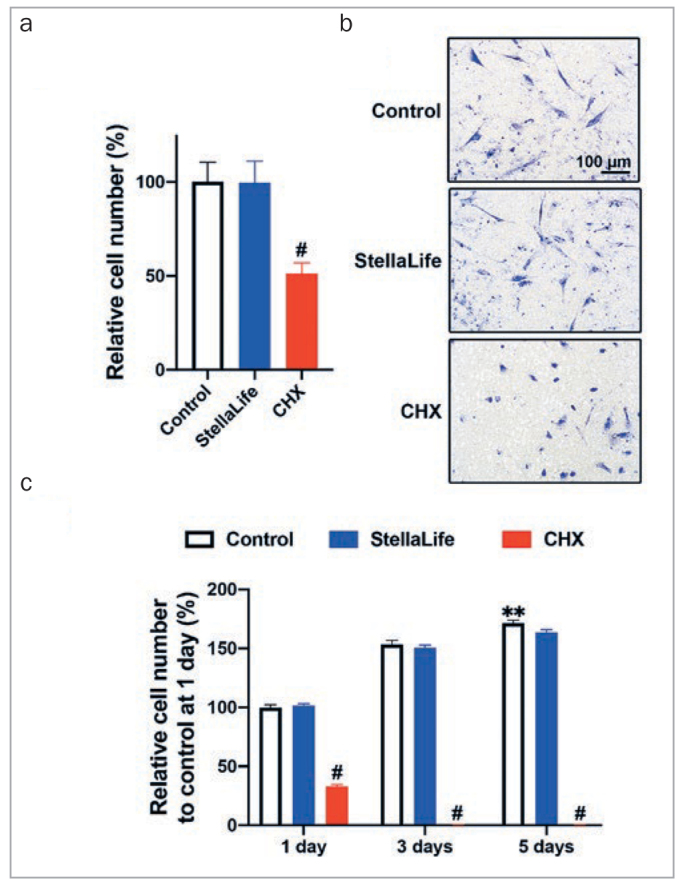
The effects of antiseptic oral rinses on HGF-1 (a, b) cell migration and (c) proliferation. (a) The migrated cell numbers and (b) images of the migrated cells at 24 h after either 1% StellaLife or CHX treatment. (c) Proliferation assay of HGF-1 cells exposed to either 1% of StellaLife or CHX at 1, 3 and 5 days. 1% StellaLife treatment showed similar cell migration and proliferation properties to control, while 1% CHX treatment inhibited cell migration and proliferation. **Denotes statistically significantly higher than all other treatment modalities. *Denotes statistically significant difference between groups. #Denotes statistically significantly lower than other modalities, p<0.05. Reprinted with permission from Fujioka-Kobayashi (2020).

Fujioka-Kobayashi et al^23^ then compared StellaLife and CHX for their ability to impact cell migration and proliferation (Fig 5). The 1% of the initial CHX concentration inhibited cell migration by roughly 50% when compared to control (Fig 5a). In contrast, cells cultured with 1% StellaLife did not negatively impact cell migration (Figs 5a and 5b). In line with these findings, a proliferation assay demonstrated that CHX treatment significantly decreased cell numbers at 1, 3 and 5 days when compared to those of the control and StellaLife groups (Fig 5c). It is noteworthy that no cells survived in CHX even at 1% concentrations, with no ability to proliferate at either 3 or 5 days (Fig 5c). An eariler study^8^ also found that StellaLife was not cytotoxic to human gingival fibroblasts and did not affect their proliferation at any dilution.

**Fig 6:**
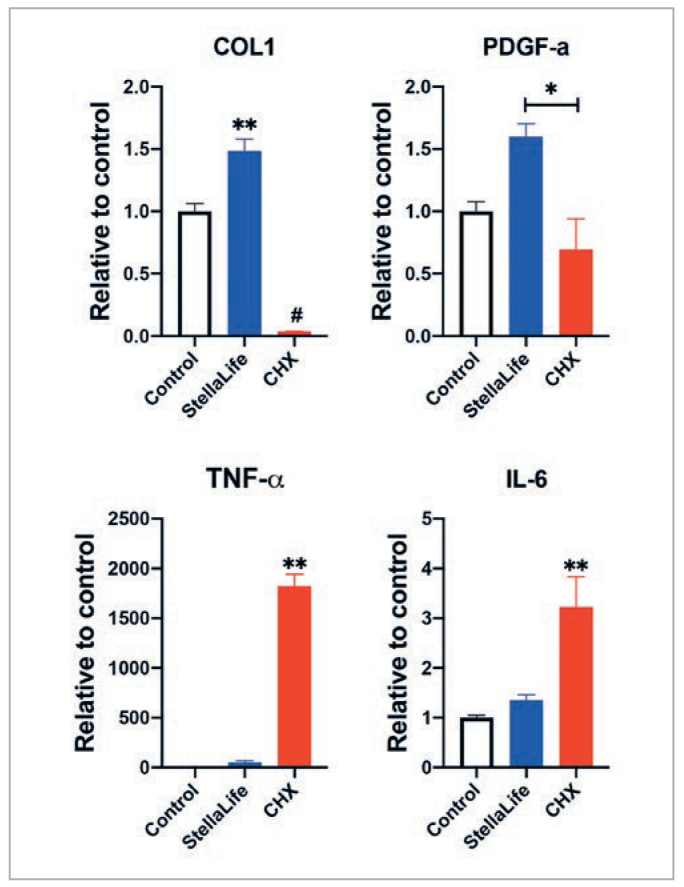
Real-time PCR of HGF-1 cells cultured in either 1% StellaLife or CHX at 5 days displaying mRNA levels of COL1, PDGF-a, TNF-α and IL-6. **Denotes statistically significantly higher than all other treatment modalities. #Denotes statistically significantly lower than other modalities, p<0.05. Reprinted with permission from Fujioka-Kobayashi, 2020.

In a final experiment,^23^ both StellaLife and CHX were compared for mRNA levels of pro-regenerative molecules COL1 and PDGF as well as inflammatory markers TNF-α and IL-6 (Fig 6). While collagen gene expression was increased by roughly 50% in the StellaLife group, they were markedly reduced in the CHX group. Furthermore, an approximately 2000-fold increase in inflammatory markers was found in the CHX group when compared to StellaLife or controls (Fig 6). These findings highlight the negative impact of CHX on gingival fibroblast activity.^23^ However, while CHX increases the amount of inflammatory cytokines in-vitro, inflammation is a part of initial phase of wound healing. Further human studies are necessary in order to more accurately evaluate how the discrepancy of inflammatory markers between CHX and StellaLife affects wound healing.

**Fig 7:**
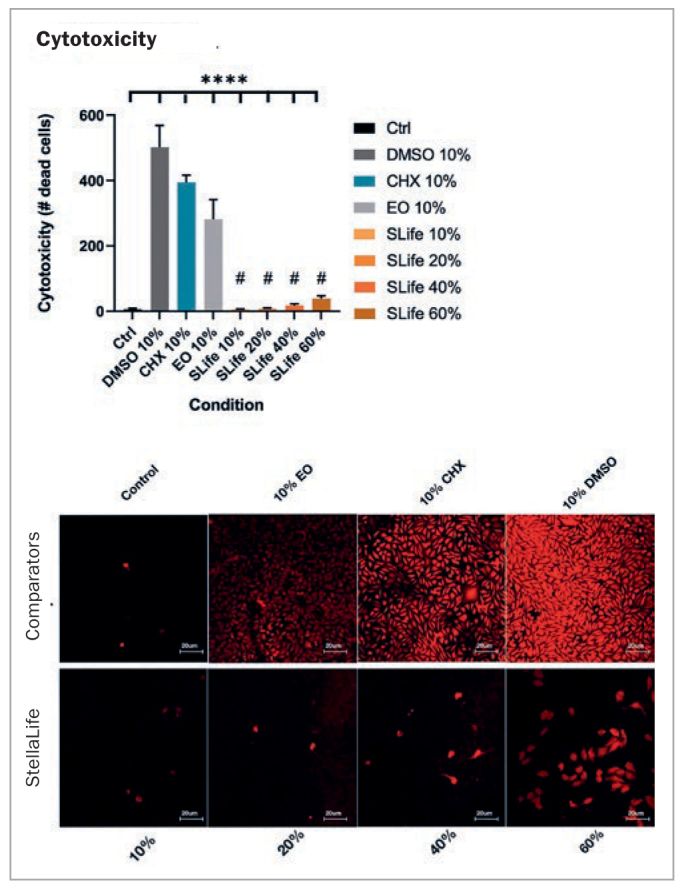
The number of dead cells taking up Zombie red dye were counted after 5 min of incubation with either of the oral rinses. Fluorescent images demonstrated limited toxicity of SLife (StellaLife) even up to 40% v/v concentrations as compared to the pronounced cytotoxicity of active comparators at 10% v/v dilutions. ****Designates highly statistically significant difference (p < 0.001) vs control. #Designates statistically significant difference (p < 0.05) vs active group comparators. Reprinted with permission from Zhou et al, 2021.

**Fig 8:**
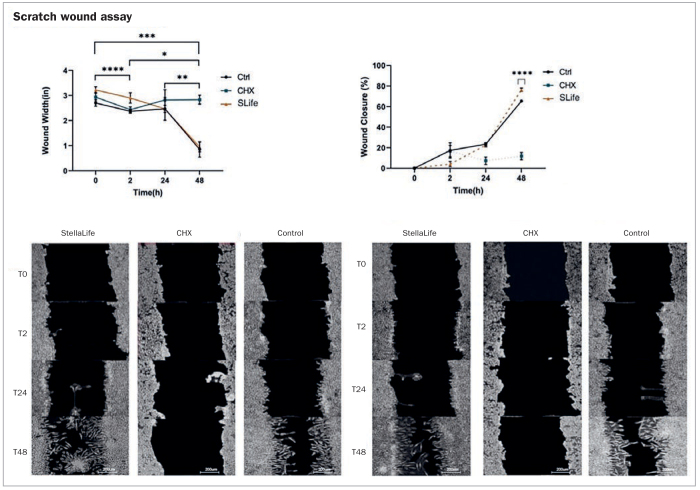
Images of the scratch wounds at two representative regions of interest for each of three conditions are shown at various timepoints following initial incubation (0 h, 2 h, 24 h, and 48 h). The StellaLife group was compared to the active comparator CHX as a positive cytotoxic control and against a media-only control to monitor wound healing in the absence of external factors. Due to cytotoxicity, the CHX group showed a wound that remained relatively the same size throughout the assay with exfoliation of cells noted at 2–24 h, which hindered cell migration. StellaLife led to a slightly more rapid wound closure compared to the control, although this was not statistically significantly different. Asterisks designate statistically significant differences, p < 0.05. Ascending numbers of asterisks are used to differentiate comparisons between increasing timepoint intervals. Reprinted with permission from Zhou et al, 2021.

**Fig 9:**
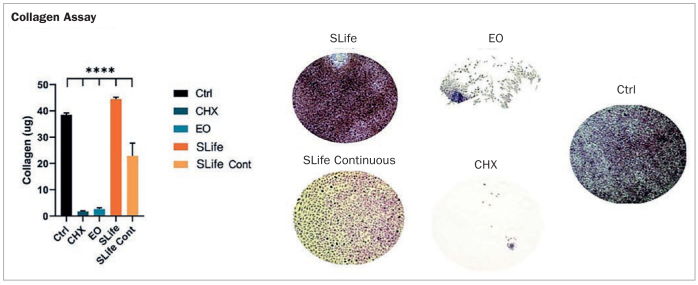
Representative samples showing total collagen (ruby red stain color) deposition in each group. Areas of cell exfoliation appear as white background. ****Designates highly statistically significant difference (p < 0.001) vs control. Reprinted with permission from Zhou et al, 2021.

Additionally, in a study performed by Zhou et al,^71^ StellaLife was compared to CHX and a commonly used essential-oil (EO)-based antiseptic rinse in terms of their effects on fibroblasts and primary oral stem cells of the apical papilla (SCAPs). Cells were compared for their cytotoxic susceptibility to each antiseptic rinse and its effects on wound healing in-vitro. A scratch-wound assay was performed and collagen deposition was investigated in-vitro. In the first experiment, it was revealed that CHX at a concentration as low as 10% of normal clinical concentrations produced a more than 400-fold increase in cell death when compared to StellaLife. When assessing the concentration-dependent cytocompatibility of StellaLife, it was found that concentrations of up to 20% v/v of StellaLife did not alter cell death. Even at concentrations of 40%–60%, the cytotoxicity of StellaLife was dramatically lower than that of 10% CHX or EO in fibroblast culture media (Fig 7). These findings highlight the much greater biocompatibility of StellaLife than CHX. Thereafter, using an established scratch-wound healing model, the migration of cells toward the leading edge of the scratch was investigated (Fig 8). When adding CHX, cells at the wound edge demonstrated cell death by 24 h and led to no increase in the wound margin by 48 h. Conversely, wound closure was observed by 24 h in the StellaLife group, which continued to close by 48 h. Thus, these findings demonstrate convincingly that while CHX actually retarded healing, StellaLife had the ability to improve healing over time. This is further exemplified in Fig 9, demonstrating that StellaLife statistically significantly facilitated greater collagen deposition (approximately 40-fold) when compared to CHX.^72^

## Clinical Uses of Stellalife

The most researched biological property of StellaLife Oral Care Recovery Kit in a clinical setting is its ability to decrease post-operative discomfort and improve pain management.^38,68^ This has been achieved through pre-emptive analgesia in which the surgical area is desensitized prior to surgical intervention.^37^ In a prospective pilot study by Lee and Suzuki,^37^ 34 patients undergoing block-graft surgery were given two different pain management regimens. The opioid-free group (14 patients) received the StellaLife Recovery Kit (StellaLife), while the control group (20 patients) were prescribed routine opioids (5 mg hydrocodone/325 mg acetaminophen). The StellaLife group was also given five tablets of “rescue” opioids. Patients were evaluated using a 10-point pain intensity scale (PIS) at 4, 8, 12, 16, and 24-hour time intervals over the following 3 days post-surgery. The StellaLife group reported a lower pain intensity than did the opioid group at every time interval investigated. Interestingly, the most noticeable difference was observed at the 2- and 4-h time intervals, when pain was most severely elevated in the opioid group. The average pain intensity score for the StellaLife group was 5.6, while the opioid group reported an average score of 7.1. The median time interval for the first “rescue” opioid in the StellaLife group was 4.3 h after surgery. Given that the most severe pain is commonly reported between 2 and 4 h after surgery, this study suggested the ability for the StellaLife Recovery Kit to significantly reduce pain post-surgery and ultimately lower the amount of opioids prescribed in private practice. However, it is important to note that taking a combination of over-the-counter drugs, specifically acetaminophen and ibuprofen in combination, may have better analgesic properties than opioids.^22,45^ Thus, it is difficult to gauge the effect StellaLife had in reducing pain, since patients were instructed to utilize it in combination with these over-the-counter analgesics.

In a retrospective cohort study by Tatch,^68^ patients were evaluated for their opioid use 1 year before and 2 years after the StellaLife Recovery Kit was incorporated into the practice’s surgical protocol. Out of 2016 patients seen prior to the new, non-opioid protocol, 58.7% were given strong opioid prescriptions. Out of 2005 patients in the first year after the new protocol implementation, only 44.8% required opioids. In the 2nd year, out of 2034 patients only, 19% of patients required strong opioids. There was no difference in the number or type of procedures performed in the office. This study successfully demonstrated how a significant drop in opioid prescriptions can occur with implementation of the StellaLife Recovery Kit in the office protocol.^68^ It is important to note that this study retrospectively evaluated the opioid prescribing routine of this particular office and does not report pain level of patients. While it is suggested that the incorporation of StellaLife led to a decrease in opioid prescription, there are other variables such as the increased criticisms of dentists overprescribing opioids.

**Fig 10:**
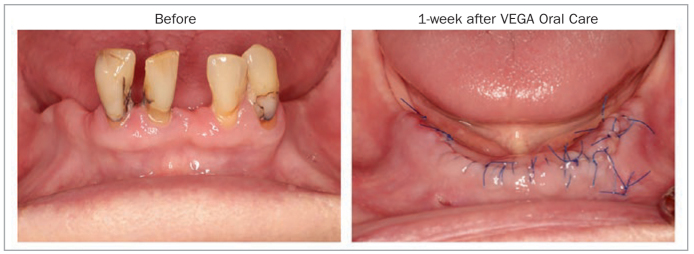
Immediate pre-operative photo and 1-week follow up of extractions with immediate implant placement. Photo courtesy of Dr. Steve Rasner.

While clinical studies are just commencing regarding the use of StellaLife, several clinical indications have been successfully implemented over the past few years. Since StellaLife is known to improve fibroblast activity, especially when compared to CHX, it opens many new possibilities regarding its use in dentistry. One of the most common indications for the StellaLife Recovery Kit is following tooth extraction. Figure 10 demonstrates a patient requiring extraction of four remaining mandibular incisors with 4 implants placed. StellaLife was given as an Oral Care recovery kit with no opioids prescribed.

**Fig 11:**
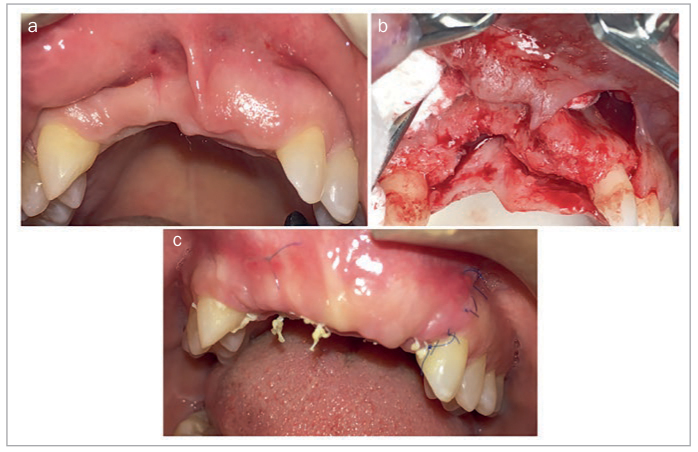
Vertical and lateral ridge augmentation in the maxillary anterior (a) pre-operatively. (b) intra-operative photo to demonstrate the extent of the defect. (c) 1 week post-operatively. Photo courtesy of Dr. Walter Tatch.

**Fig 12:**
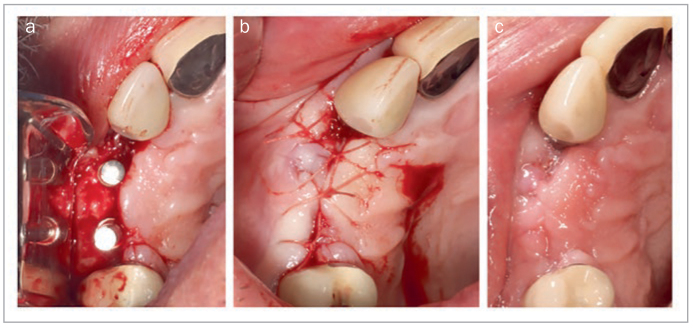
Implant placement #5, #6 (American numbering). (a) Flap reflection with initial drills at #5, #6 (American numbering). (b) Immediate closure using simple interrupted sutures. (c) 2 weeks post-operatively. Photo courtesy of Dr. Nathan Estrin.

**Fig 13:**
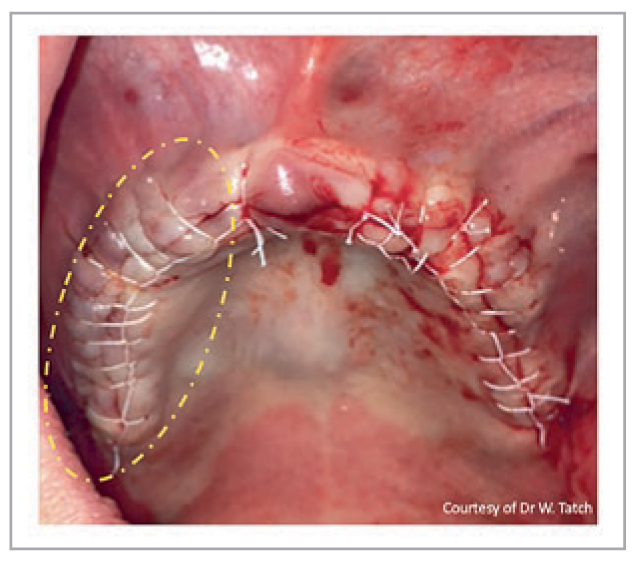
Full implant rehabilitation case in the maxillary arch. StellaLife gel was applied immediately post-operatively only on the right side. Clinical photo was taken 40 min after gel application. Photo Courtesy of Dr. Walter Tatch.

Similarly, StellaLife has been frequently employed during ridge augmentation and dental implant placement. Figure 11 shows lateral and vertical ridge augmentation in a severely deficient anterior maxilla. The recovery kit was utilized starting 3 days prior to procedure and continued post-operatively. At 1 week post-operatively, minimal inflammation was seen in the area and no plaque accumulation was observed. Figure 12 presents a 67-year-old patient who was referred for implant therapy at sites #5 and #6 (American numbering system; FDI #14 and #13). During implant placement, incorporation of StellaLife Recovery Kit was utilized. Accelerated healing was observed at two weeks with minimal inflammation observed and no plaque accumulation (Fig 12b). Additionally, Fig 13 represents a full implant rehabilitation case in the maxillary arch. StellaLife gel was applied immediately post-operatively only on the right side. After 40 min, an appreciable difference in post-operative inflammation can be observed in this split-mouth case. While the right side appears less inflamed, conclusions from this single split-mouth case cannot be reached due to potential cross-over effects of the StellaLife gel. Additionall research is necessary to evaluate the localized effects of the gel and the validity of split-mouth studies in this approach. In our office, the StellaLife gel is applied to the surgical sites immediately post-operatively and patients are instructed to apply the gel last, to avoid washing it away with the rinse.

**Fig 14:**
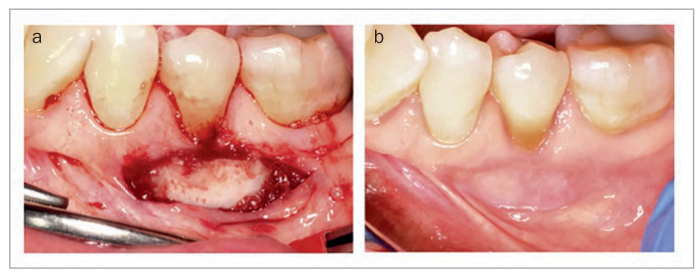
(a) Immediate post-operative photo of a free gingival graft fixated to recipient site #20 utilizing periacryl oral tissue adhesive. (b) 2 weeks post-operatively. Photo courtesy of Dr. Nathan Estrin.

Additionally, free gingival grafts are good indications for the StellaLife Recovery kit, due to the healing of secondary intention at both the donor and recipient sites. Patients have reported a therapeutic effect in our office when placing the gel over the donor site on the palate. Figure 14 demonstrates a patient referred for inadequately attached gingiva #20 (American tooth numbering system; FDI #35). The patient elected to have a free gingival graft rather than connective tissue graft after discussing both options. The recipient was bed-prepared utilizing a #15 blade. Epithelialized tissue was harvested from the patient’s palate and fixated to the recipient site utilizing periacryl oral tissue adhesive (Fig 14a). The graft was immobilized before the patient was released. At 2 weeks post-operatively, accelerated would healing was observed with no inflammation present (Fig 14b).

**Fig 15:**
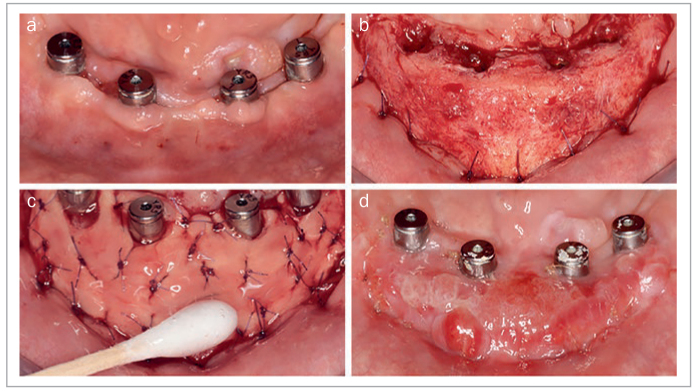
(a) Pre-operative photo showing 4 mandibular implants with a lack of attached tissue. (b) Recipient site preparation utilizing a split-thickness incision and an apically positioned flap. (c) Acellular Dermal Matrix (Alloderm). (d) 3-week post-operative photo. Photo courtesy of Dr. Michael Pikos.

As an alternative to free gingival grafts, acellular dermal matrix is an alternative frequently used for augmenting soft tissues either around or prior to dental implants.^52^ Figure 15 highlights the use of StellaLife in a vestibuloplasty with acellular dermal matrix, utilized to alter the patient’s phenotype and thicken the attached tissue around existing implants in the mandible. In Fig 15c, StellaLife gel was applied to the surgical area. At three weeks post-operatively (Fig 15d), a change in the patient’s phenotype was observed.

**Fig 16:**
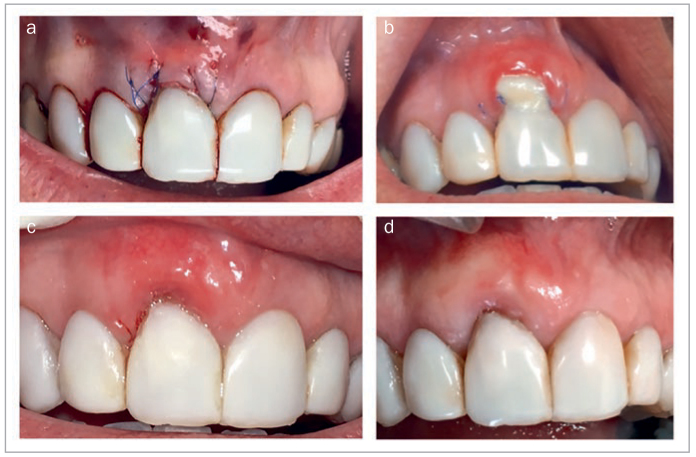
Utilization of StellaLife Recovery Kit for management of post-operative soft tissue dehiscence. (a) Immediate post-operative photo of recession coverage #8 utilizing Alloderm with tunneling technique. (b) Soft tissue dehiscence. (c) 10 days post-operatively after utilizing recovery kit. (d) 3 weeks post-operatively.

StellaLife has also been employed to manage soft-tissue dehiscence as a post-operative complication. In Fig 16, Alloderm was used for recession coverage at site #8 (FDI: #11) via the tunneling approach. The patient was pulling at the Alloderm post-operatively because she thought “it was not supposed to be there.” In Fig 16b, a soft-tissue dehiscence can be seen post-operatively. After applying the recovery kit, the patient presented with soft-tissue coverage at the 10-day follow-up (Fig 16c) with continued tissue maturation at the 3-week follow-up (Fig 16d).

**Fig 17:**
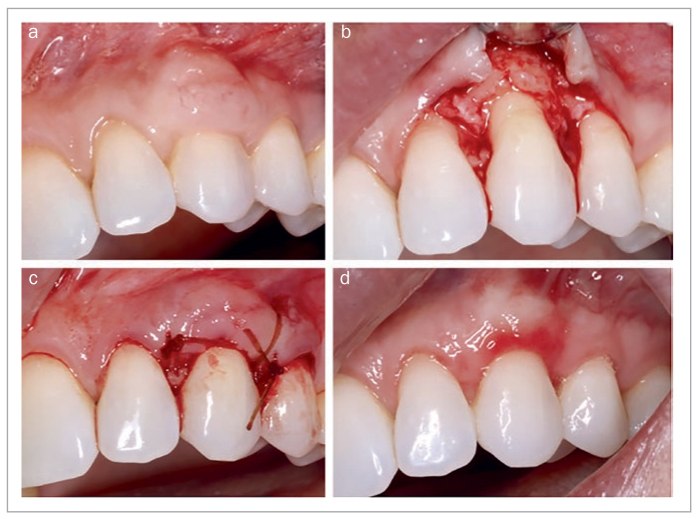
(a) Pre-operative view of a lesion later diagnosed as a peripheral ossifying fibroma. (b) An internal bevel incision was utilized to remove the excess tissue (before placing it in formalin for biopsy) and ostectomy was performed. (c) Immediate post-operative photo where external vertical mattress sutures were utilized to apically position the flap. (d) 2 weeks post-operatively after the patient was instructed to rinse with StellaLife daily. Photo courtesy of Dr. Nathan Estrin.

Figure 17a depicts a lesion later diagnosed as a peripheral ossifying fibroma. An internal bevel incision was made to remove the excess tissue and ostectomy was performed (Fig 17b). External vertical mattress sutures were placed to apically position the flap (Fig 17c). Figure 17d shows the 2-week post-operative situation, after the patient was instructed to rinse with StellaLife.

While future randomized controlled clinical trials and in-vivo studies remain necessary in order to further evaluate the accelerated healing aspects, antimicrobial, and anticancer resistance, several practitioners have reported substantial improvements in wound healing post-surgery when utilizing StellaLife.

## Discussion

The opioid crisis, especially in the US, has become a public health crisis. Particularly US clinicians have been over-prescribing, which has subsequently created a drug overuse problem (most pronounced among teenagers), leading to a dramatic increase in fatal overdoses.^55^ More than 5 years have passed since the Food and Drug Administration (FDA), National Institute of Drug Abuse (NIDA), National Institutes of Health (NIH), Drug Enforcement Agency (DEA), the Centers for Disease Control (CDC), and medical and dental organizations such as the American Medical Association (AMA), American Dental Association (ADA)4, and the American Association of Oral and Maxillofacial Surgeons (AAOMS) collectively declared a drastic need to combat their misuse.^4,12,14,20,32^

Opioids are commonly prescribed by dentists after oral surgery for the management of acute pain, but doubt has been cast upon their use in recent years, given the number of teens who have reported that their drug abuse/addictions to other narcotics often began with opioids prescribed by healthcare providers. Since the mid 1990s, deaths caused specifically from opioid overdose has more than quadrupled, which precisely parallels the increase in opioid prescriptions written in dental and medical practices.^48^^,54^ Amazingly, some countries have entirely banned opioid use in dental practice and their ability to control pain has been manageable.

Given that the location of surgery is known prior to the injury, pre-emptive analgesia is a tool that can be implemented to help clinicians reduce the amount of opioid use in a private practice setting.^37,68^ Active ingredients in StellaLife such as *Arnica montana*, chamomile (*Matricaria recutita*), and *Aconitum* are all natural alternatives for pain management with an extended history of use for such purposes.^5,44,64^ As discussed in this review article, various in-vitro and clinical studies have demonstrated the retention of these analgesic properties in the final StellaLife composition, and the final StellaLife Recovery Kit has successfully been applied to reduce the frequency of opioids prescribed in private practice.^37,68^ However, it is necessary to conduct additional studies in which StellaLife is the only analgesic used. While a main mechanism of action is believed to be through pre-emptive analgesia and post-operative fibroblast activity improvements, pre-clinical in-vitro studies have validated clinical observations by demonstrating better biocompatibility of StellaLife when compared to CHX. There is still much research needed to fully understand the analgesic properties of StellaLife and better address how the combination of the active ingredients in StellaLife works in synergy to improve clinical outcomes.

While scratch-wound assays of *Plantago* and *Arnica montana* have individually demonstrated accelerated healing,^42,72^ Zhou et al^71^ demonstrated that these same properties were also maintained and potentially improved in the final formulation of StellaLife. Furthermore, when compared to CHX, StellaLife demonstrated dramatic improvements in wound healing in-vitro. However, inflammation is normal in the intial phase of wound healing. Additionally, cytotoxicity may be desired in certain clinical situations, e.g. when dealing with post-operative infection. Further human studies remain necessary to confirm the accelerated healing with StellaLife and better understand its mechanism of action, as well as to directly compare it with chlorhexidine.

StellaLife also possesses antibacterial properties with several active ingredients, including *A. indica*, *Calendula*, and *Echinacea*.^21,35,63^ Randomized clinical trials by Khairnar et al^35^ and Botelho et al^11^ displayed their effects in reducing gingivitis clinically using *Calendula* and neem, respectively. However, there are no clinical trials demonstrating the anti-gingivitis effects of StellaLife and its potential as an adjunctive rinse in the management of periodontitis. Given how *A.* *indica, Plantago,* and other active ingredients limit several pro-inflammatory cytokines associated with periodontitis and interrupt the NF-κB pathway, further research on StellaLife’s combined ingredients are thus warranted.^15,26^^,28,30,35^ Additional research is required to evaluate StellaLife’s effect on resilient biofilms and periodontitis.

Additionally, one of the main ingredients in StellaLife, *A. indica*, has been demonstrated to possess anti-cancer properties by inhibition of the NF-κB pathway, reduction in carcinogenic cytokines, and restoring balance in the Bcl-2:Bax ratio.^28,41^ Specifically in dental medicine, a growing number and incidence of oral cancers have been reported over the past decade.^17^ Thus, utilizing Stellalife Oral Rinse may be advantageous, particularly after cancerous lesion removal, though much further research addressing this topic is needed. Both pre-clinical (in-vitro and animal research), as well as clinical studies are necessary to evaluate these anti-cancerous properties.

## Conclusion

This review article investigated the active ingredients in StellaLife, highlighting its potential as an antimicrobial and anticancer agent that also accelerates wound healing and offers superior pain management. Its ability to manage pain through pre-emptive analgesia has been demonstrated in human studies, resulting in a reduction in opioid use. StellaLife has also shown superior biocompatibility and wound healing potential when compared specifically to chlorhexidine. StellaLife demonstrated the potential to be selectively cytotoxic towards bacteria while remaining harmless or even beneficial towards human cells, including epithelial cells and gingival fibroblasts. Despite these promising early results, additional clinical research is warranted with much future potential for use as a safer and more effective oral rinse post-surgery when compared to CHX.
